# α‐Adrenergic receptor blockade attenuates pressor response during mental stress in young black adults

**DOI:** 10.14814/phy2.14642

**Published:** 2020-12-23

**Authors:** Jin Hee Jeong, Michelle L. Brown, Gaston Kapuku, Gregory A. Harshfield, Jeanie Park

**Affiliations:** ^1^ Department of Medicine Georgia Prevention Institute Medical College of Georgia Augusta University Augusta GA USA; ^2^ Division of Renal Medicine Department of Medicine Emory University Atlanta GA USA; ^3^ Department of Veterans Affairs Health Care System Decatur GA USA

**Keywords:** Blacks, Hypertension, Mental Stress, α‐Adrenergic Receptor Blocker

## Abstract

Black individuals exhibit increased blood pressure (BP) responses to sympathetic stimulation that are associated with an increased risk of hypertension (HTN). We tested the hypothesis that α_1_‐adrenergic blockade inhibits the increased BP response during and after 45‐min stress in young normotensive Black adults, which may be mediated, in part, by dampened vasoconstriction and decreased renal sodium retention. Utilizing a double‐masked randomized, crossover study design, 51 normotensive Black adults (31 ± 8 yr) were treated with either a placebo or 1 mg/day of prazosin for 1 week. On the final day of each treatment, hemodynamic measures and urinary sodium excretion (UNaV) were collected before (Rest), during (Stress) and after (Recovery) 45 min of mental stress induced via a competitive video game task. During the Stress period, diastolic BP and total peripheral resistance (TPR) were significantly lower with prazosin compared to placebo (*p* < .05 for both). Similarly, we observed lower systolic BP, diastolic BP, and TPR during the Recovery period with prazosin versus placebo (*p* < .05 for both). There was no effect of prazosin on stress‐associated UNaV. The change in systolic BP from Rest to Recovery was positively associated with the change in TPR with both treatments (*p* < .05 for both). In summary, prazosin treatment dampened BP reactivity to 45‐min mental stress and lowered post‐stress BP over the recovery period, which was linked to reduce TPR in young normotensive Black adults. These results suggest that α_1_‐adrenergic receptor activity may contribute to BP responses and delayed BP recovery to prolonged mental stress through increased vasoconstriction in Black adults.

## Graphical abstract

Psychological stress contributes to the susceptibility and severity of hypertension. Black individuals are known to elicit hyper‐hemodynamic responsiveness to various stressors. The present study demonstrated that the blockage of alpha‐adrenergic receptor activity improved the blood pressure responses to mental stress in young normotensive Black adults.

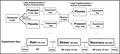

## 1 INTRODUCTION

Black individuals have an increased risk of incident hypertension (HTN), as well as poorer rates of blood pressure (BP) control after the onset of HTN (Howard et al., 2013; Virani et al., 2020). Accumulating evidence suggests that psychological stress contributes to the pathogenesis of HTN (Spruill, 2010; Treiber et al., 2003). Augmented BP reactivity to mental stress and delayed BP recovery back to pre‐stress levels are associated with increased risk for the development of HTN in healthy normotensive adults (Markovitz et al., 1998; Steptoe & Marmot, 2005). In addition to higher levels of reported environmental and social psychological stress (Calhoun, 1992), Black individuals have exaggerated hemodynamic reactivity to a variety of stressors (Light et al., 1993; Murphy et al., 1991). However, the precise mechanisms of increased stress‐induced BP in Black adults are poorly understood.

The sympathetic nervous system (SNS) plays a key role in the regulation of BP and cardiovascular (CV) reactivity to stress. The SNS responses to stress include the release of norepinephrine and epinephrine, which stimulates adrenergic receptors in various organs and the vasculature to increase BP. Increased adrenergic reactivity to an acute stressor has been reported in high‐risk normotensive populations including Black individuals (Julius & Nesbitt, 1996; Noll et al., 1996). In particular, young Black adults have been found to have an increased degree of vasoconstriction in response to a given increase in sympathetic activation. Black adults have greater vasoconstriction in response to infusion of the α_1_‐adrenergic agonist, phenylephrine (Adefurin et al., 2013; Sherwood & Hinderliter, 1993; Stein et al., 2000), or in response to spontaneous bursts of muscle sympathetic activity, that is, neurovascular transduction (Vranish et al., 2018), that contributes to an increased pressor response to stress by inducing greater peripheral vascular constriction (Brownlow et al., 2020; Okada et al., 2016; Stein et al., 2000; Vranish et al., 2018). In addition, previous evidence has shown that Black individuals have exaggerated BP responses, during short acute sympathetic stimulation (<10 min) induced by the cold pressor test, intravenous administration of α‐adrenergic receptor agonists (Adefurin et al., 2013; Stein et al., 2000) or a video game (Murphy et al., 1986, 1994) although some reported no racial difference in BP response to different types of psychological stress (McAdoo et al., 1990). However, whether BP reactivity and post‐stress recovery to a prolonged and low‐level mental stress that may be more relevant to real‐life circumstances are mediated by increased vasoconstriction due to α‐adrenergic activation in Black individuals is unknown.

Black individuals are also characterized by altered body sodium regulation in relation to the resultant BP change (Svetkey et al., 1996). We previously demonstrated that individuals without an adequate compensatory increase in the urinary sodium excretion in response to a stress‐induced increase in BP (i.e., impaired stress‐induced pressure natriuresis) showed a delayed BP recovery at least in part due to increased blood volume (Snieder et al., 2002), which may contribute to the premature development of HTN (Ge et al., 2009; Harshfield et al., 2002a; Hur et al., 2014). In addition to the high prevalence of salt sensitivity (i.e., excessive BP increase to salt intake) (Svetkey et al., 1996), our previous reports demonstrated that a large proportion of Black individuals have a diminished natriuretic response to increases in BP during stress (Harshfield et al., 2007; Harshfield, et al., 2002). In animal models with inherited or acquired predisposition for HTN, exposure to environmental stress provokes sodium retention associated with increased renal sympathetic activity (Koepke et al., 1988; Lundin & Thoren, 1982) possibly via adrenoreceptor‐mediated increases in sodium reabsorption (Graham et al., 1982). However, the underlying mechanisms of impaired stress‐induced pressure natriuresis in Black adults remain poorly understood. The overall goal of this study was to assess the effects of an α_1_‐adrenergic receptor blockade on BP response and sodium handling before, during, and after an acute prolonged bout of mental stress in Black adults. We hypothesized that stress‐evoked peripheral vasoconstriction and increased renal sodium retention contribute to an increase in BP during mental stress, which is in part mediated by α_1_‐adrenergic receptor activity in Black adults. α‐blockers as an antihypertensive medication are generally prescribed as a third or fourth‐line agent due to its modest BP‐lowering effects and adverse side effects (Heran et al., 2012); the α‐blocker prazosin, however, was used to determine the effect of the α‐adrenergic blockade on hemodynamic, vascular, and renal responses to stress in Black adults.

## 2 METHODS

### 2.1 Study participants

**Figure 1:**
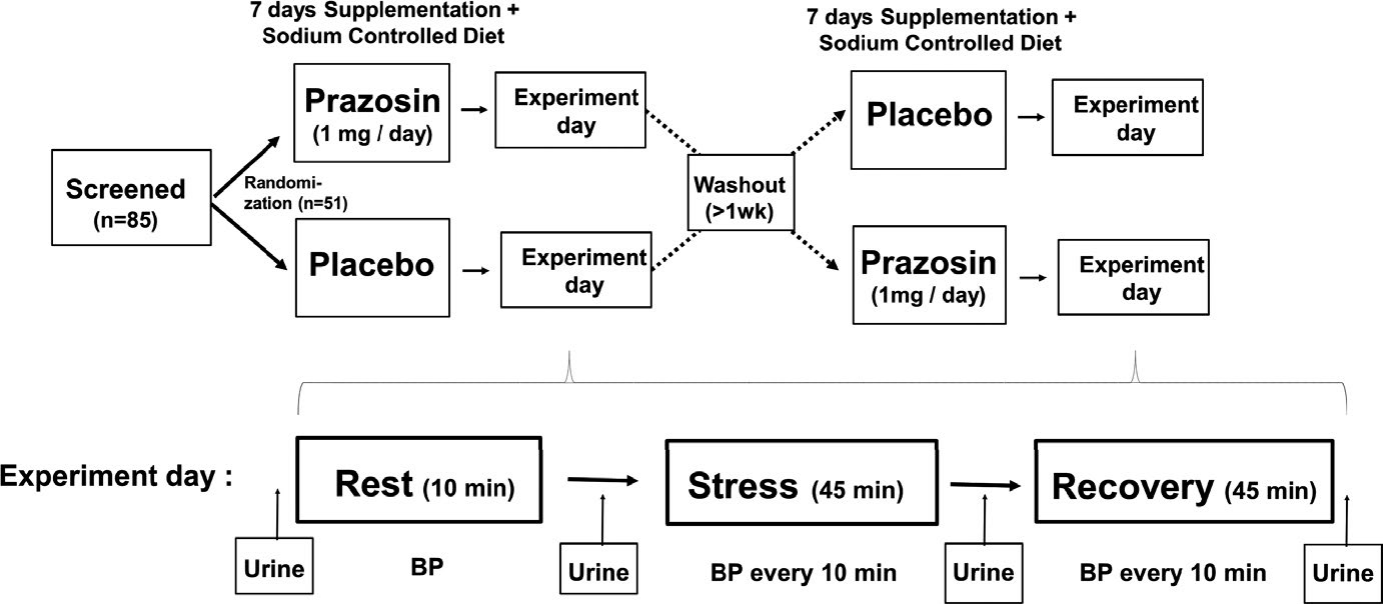
Study Design. BP: Blood Pressure

Among a total of 85 participants screened, 51 participants met the inclusion/exclusion criteria and completed the study protocol between 2015 and 2019 (Figure *1*). Inclusion criteria included: AA by self‐report, 18 to 50 years old, and in overall good health. Exclusion criteria included: use of medications that influence BP, pregnancy, and presence of chronic disease conditions including diabetes, coronary artery disease, and heart failure. Informed consent was obtained from all participants prior to any measurements and a brief physical exam was performed by a physician prior to testing to determine eligibility. All procedures were approved by the Institutional Review Board of Augusta University in accordance with the institutional guidelines and registered with clinicaltrials.gov (NCT02431936).

### 2.2 Study design

This study was a randomized, double‐masked, placebo‐controlled, crossover trial aimed to determine the effects of prazosin (α_1_‐adrenergic receptors blocker) on hemodynamic and natriuretic responses during mental stress in Black adults. Participants were randomly assigned to receive either placebo or prazosin (α_1_‐adrenergic receptor blocker, 1 mg by mouth in the morning daily) for one week during which participants were on a fixed sodium‐controlled diet to ensure similar levels of sodium balance at the time of testing. With guidance from a nutritionist who identified the sodium content of selected foods, participants selected their own diet to equal 4,000 ± 200 mg of sodium per day, which has been used in our previous studies to examine pressure natriuresis response to mental stress in Black individuals (Ge et al., 2009; Harshfield, et al., 2002; Jeong et al., 2019). A week of daily administration of 1mg prazosin was chosen to ensure the full drug effect on the testing day while avoiding adverse effects, such as “first‐dose effect” given to normotensive individuals by a more prolonged duration with a lower dose of 1 mg. Overnight urine samples were collected the night prior to the participants testing to assess dietary compliance. This method has been shown to successfully reduce the variability of sodium intake, as estimated by overnight sodium excretion in free‐living individuals (Savoca et al., 2007). On the final day of treatment, the participant performed the stress test protocol described below. This was followed by a one to two‐week washout period. The subject then performed the same procedure while on the second treatment (Figure *1*). Randomization of treatment (placebo versus. prazosin) was performed and maintained by the university pharmacy. Participants were asked to refrain from drinking caffeinated or alcoholic beverages on the day before the studies.

### 2.3 Stress testing protocol

The mental stress protocol was a modified version of the standard stress testing protocol we have used successfully in over 1,000 participants as described previously in both adult (Koepke, 1985) and pediatric populations(Barbeau et al., 2003; Harshfield et al., 1991, 2003; Harshfield, et al., 2002). The stressor was a competitive video game task played against another individual for monetary reward. On the testing day, the participants were tested in a private room in a comfortable chair. The participants took their last dose of the study drug immediately before the stress testing. The testing period included: 10 min of rest (Rest), 45 min of mental stress (Stress), in which the participants played a racing or sports video game against one another for a cash prize (Nintendo Wii Mario Kart), followed by another 45 min of rest (Recovery). The participants watched a movie that did not contain stimulating scenes during the Rest and Recovery periods.

### 2.4 Measurements

BP was monitored by an automated BP machine (Dinamap Model 1864 SX, Critikon Inc.) Thoracic bioimpedance (NCCOM‐3, Bo Med Medical Manufacturing Ltd) was used to measure stroke volume (SV), heart rate (HR), and cardiac output (CO). Total peripheral resistance (TPR) was calculated as [(diastolic BP + 1/3 (systolic BP – diastolic BP)]/ CO. Measurements were taken at 10‐min intervals throughout the testing period. Spot urine samples were obtained before and after the Rest, Stress, and Recovery periods. The participants were required to drink 200 ml of water every hour to ensure that they remained hydrated and were able to provide adequate urine samples. Volume corrected urinary sodium excretion (UNaV) was analyzed by the NOVA 16 Analyzer (NOVA Biomedical, Waltham, MA) that uses an ion‐selective electrode technique. The intra‐assay coefficient of variation for UNaV was < 3% and the inter‐assay coefficient was 4%.

### 2.5 Power analysis

A power analysis was conducted using estimates from relevant studies that found a significant difference in systolic BP response to mental stress after treatment with an α_1_‐adrenergic receptor blocker (12.2 ± 5.7 versus. 5.7 ± 6.0 mmHg, *p* < .01) in hypertensive individuals (Lee et al., 1996). Our previous study showed a significant increase in stress‐induced UNaV after treatment with an angiotensin receptor blocker (0.05 ± 0.13 versus. 0.09 ± 0.017 mEq, *p* < .05) in normotensive Black individuals (Jeong et al., 2019). Based on these results, enrolling 51 participants had > 80% power with an α of 0.05 to detect a treatment effect.

### 2.6 Statistical Analysis

Values are presented as mean ± *SD* unless otherwise noted. Differences in participant characteristics between treatment days were determined using independent *t*‐tests. Carryover effects were examined for the potential influence of the order of treatments in our crossover design (Byron & Kenward, 2003) and no significant effects were found. Therefore, no adjustments were made in the subsequent analysis. Repeated‐measures analysis of variance (ANOVA) was used to identify the effect of treatments (placebo and prazosin) on the changes in hemodynamic variables and urinary excretion across different conditions (Rest, Stress, and Recovery) using averaged values throughout a condition and across 10‐min intervals in each condition. Bonferroni test was used for post hoc analysis when a significant main effect was found. In addition, between‐treatment comparisons were conducted to examine the difference in the absolute and the change from Rest at each measured time point since there was no difference in variables at the Rest between treatment days. The degree of association between the magnitude of changes in BP and other hemodynamic variables and UNaV was analyzed by Pearson's correlation coefficient. Statistical significance was set at an alpha level of 0.05. All analyses were performed using SPSS version 24.0 (IBM Corporation, Somers, NY).

## 3 RESULTS

### 3.1 Participant characteristics

Descriptive characteristics of the 51 participants that completed the crossover experiments without adverse events are presented in Table 1. The study group includes 52.9% of female participants.

**Table 1:** Subject Characteristics

Characteristics (*n* = 51)			*P* value
Age (yr)	31.4 ± 8.0		
BMI (kg/m^2^)	28.4 ± 9.1		
Height (cm)	169.1 ± 8.8		
Weight (kg)	78.9 ± 21.0		
Gender (male, %)	24 (47.1)		
Blood Chemistry			
Albumin (g/dL)	4.4 ± 0.3		
K (mEq/L)	4.0 ± 0.6		
Creatinine (mg/dL)	0.9 ± 0.2		
Glucose (mg/dL)	87 ± 11		
Calcium (mg/dL)	9.6 ± 0.4		
Resting Hemodynamics	Placebo	Prazosin	
SBP (mm Hg)	116 ± 10	115 ± 11	0.849
DBP (mm Hg)	66 ± 8	67 ± 9	0.489
HR (bpm)	72 ± 10	70 ± 10	0.421
SV (mL)	82 ± 20	78 ± 19	0.415
TPR (dynes·sec·cm^−5^)	15.7 ± 10.6	15.8 ± 4.0	0.983
CO (L/min)	5.9 ± 1.5	5.5 ± 1.1	0.130
UNaV (mEq/min)	0.07 ± 0.06	0.07 ± 0.04	0.581

Note

Values are reported as mean ± standard deviation. BMI, Body Mass Index; bpm, Beat Per Minute; CO: Cardiac Output; DBP, Diastolic Blood Pressure; HR, Heart Rate; SBP, Systolic Blood Pressure; SV, Stroke Volume; TPR, Total Peripheral Resistance; UNaV, Urinary Sodium Excretion Rate.

### 3.2 The hemodynamic responses to stress with and without prazosin

There was no difference in any of the hemodynamic variables at Rest between placebo and prazosin treatment (*Table* *1*). There was a significant Treatment (placebo versus. prazosin) x Time (Rest, Stress, Recovery) interaction effect for the mean TPR (*F*_(2, 156)_ = 68.6, *p* = .006) after adjustment for baseline values. Specifically, TPR was increased in response to stress with placebo while no change in TPR was observed with prazosin. This stress‐induced elevation in TPR was maintained throughout the Recovery period with placebo, but stayed unchanged at a similar level to Rest during the Recovery period with prazosin. The mean SBP during the Recovery period demonstrated a similar nonsignificant trend with less of a stress‐induced increase and a greater drop during Recovery with prazosin compared to placebo (Treatment x Time interaction effect, *F*_(2, 190)_ = 2.52, *p* = .085).

**Figure 2:**
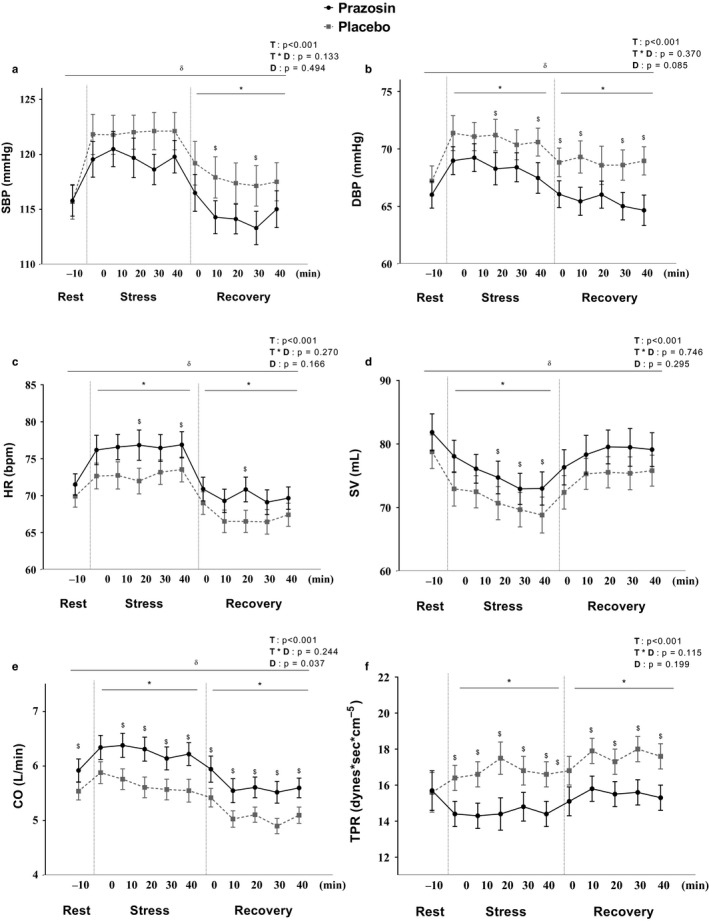
a–f. Effect of Prazosin on Stress‐induced Hemodynamic Changes. T: Time Effect, D: Drug Effect, T * D: Time x Drug Interaction Effect. δ: Significant Time effect on Rest, Stress versus Recovery (*p* < .05). *: Significant Drug effect in the mean value within the condition (*p* < .05). $: Significantly different between Drug groups at the time point (*p* < .05)

The mean DBP and TPR during the Stress period were significantly lower (68.2 ± 8.1 versus. 70.6 ± 8.3 mmHg, *p* = .015; 14.5 ± 4.0 versus 17.5 ± 6.9 dynes·sec·cm‐^5^, *p* = .001, respectively), and the mean HR and CO during Stress were significantly higher (76.8 ± 12.4 versus 72.7 ± 11.5 bpm, *p* = .023; 5.6 ± 1.3 versus 6.3 ± 1.4 L/min, *p* < .001, respectively) with prazosin compared to placebo (Figure 2). A similar trend was observed in post‐stress hemodynamics with lower mean values of SBP, DBP and TPR (114.6 ± 10.0 versus. 117.5 ± 12.2 mmHg, *p* = .042; 65.5 ± 7.9 versus. 68.7 ± 8.9 mmHg, *p* = .012; 15.5 ± 4.9 versus. 17.5 ± 5.6 dynes·sec·cm‐^5^, *p* = .003, respectively) and greater mean values of HR and CO (70.4 ± 17.1 versus. 67.4 ± 10.3 bpm, *p* = .035; 5.7 ± 1.4 versus. 5.1 ± 1.0 L/min, *p* = .001, respectively) over the course of the Recovery period with prazosin compared to placebo (Figure 2, Table *2*). The mean SBP, DBP, HR, and CO were significantly increased from Rest in response to Stress with both placebo and prazosin treatment; however, the magnitude of the stress‐induced increases in SBP, DBP, and TPR were lower with prazosin compared to placebo (*p* < .01 for all, Table 2). These stress‐induced increases in SBP, DBP, HR, and CO were normalized to baseline levels upon the cessation of the stressor with both treatment groups *(*Table 2 *)*. There was no sex difference in all hemodynamic responses to stress and prazosin.

**Table 2:** Changes in cardiovascular hemodynamic values and urinary sodium excretion rates immediately after (Stress) and 45‐min after (Recovery) mental stress from rest following Placebo or Prazosin treatment

Variable	Placebo		Prazosin		*P* value^#^		
	Stress	Recovery	Stress	Recovery	Time	Drug	T*D
ΔSBP (mm Hg)	6.0 ± 8.9*	1.5 ± 7.7	3.6 ± 6.7*	−1.2 ± 6.4	0.000	0.049	0.885
ΔDBP (mm Hg)	3.7 ± 6.5*	1.5 ± 6.8	2.5 ± 5.6*	−0.3 ± 5.6	0.000	0.195	0.730
ΔHR (bpm)	3.1 ± 7.5*	−2.8 ± 6.9*	5.3 ± 8.2*	−1.5 ± 5.9	0.000	0.201	0.496
ΔSV (mL)	−8.5 ± 9.8*	−3.6 ± 9.3*	−7.2 ± 11.1*	−3.2 ± 7.3*	0.000	0.649	0.567
ΔTPR (dynes·sec·cm^−5^)	1.0 ± 3.5*	1.9 ± 3.6*	−1.2 ± 9.8	−0.2 ± 7.1*	0.005	0.051	0.712
ΔCO (L/min)	0.1 ± 0.7	−0.4 ± 0.6*	0.3 ± 0.9*	−0.3 ± 0.6*	0.000	0.132	0.476
ΔUNaV (mEq/min)	0.09 ± 0.13*	0.10 ± 0.09*	0.10 ± 0.18*	0.09 ± 0.08*	0.940	0.816	0.383

Note

Values are mean ± *SD*. bpm, Beat Per Minute; CO, Cardiac Output; DBP, Diastolic Blood Pressure; HR, Heart Rate; SBP, Systolic Blood Pressure; SV, Stroke Volume; T * D, Time x Drug Interaction Effect; TPR, Total Peripheral Resistance; UNaV, Urinary Sodium Excretion Rate.

* Indicates difference from Rest within the treatment at α < 0.05 by paired t‐tests.

# *P* values are from repeated ANOVA tests.

### 3.3 The determinants of stress‐induced blood pressure change with and without prazosin

SBP was positively associated with TPR both with prazosin (r = 0.533, *p* = .001) and placebo (r = 0.446, *p* = .001) during Recovery. In agreement with this, positive relationships were also observed between the changes in mean SBP and mean TPR from Rest to Recovery both with prazosin (r = 0.312, *p* = .031) and placebo (r = 0.354, *p* = .013). The change in mean SBP from Rest to Stress was positively correlated with the change in mean HR over the same period with prazosin (r = 0.287, *p* = .046), but not with placebo (r = 0.163, *p* = .258).

### 3.4 The effects of prazosin on stress‐induced urinary sodium excretion

UNaV levels were similar at Rest, Stress, and Recovery between placebo and prazosin treatments (*p* > .05). In response to stress, UNaV significantly increased at a similar magnitude with prazosin and placebo (Table 2). The total overnight urinary sodium excretion collected each morning of the experimental day was similar between placebo and prazosin (114 ± 58 versus 105 ± 62 mEq, *p* = .433).

## 4 DISCUSSION

This placebo‐controlled crossover study investigated whether the α_1_‐adrenergic activity is involved in the BP response to mental stress in Black adults. We hypothesized that the activation of α_1_‐adrenergic receptors is a potential contributing factor for the increased pressor response during and after an acute bout of mental stress in Black adults and that this response is mediated via increased peripheral vasoconstriction and increased renal sodium retention. Our results provided partial support for this hypothesis. Suppression of α_1_‐adrenergic receptor activity with 7‐days of prazosin treatment dampened stress‐induced increases in BP during 45 min of mental stress and lowered post‐stress BP over the course of the 45‐min recovery period compared to placebo. This attenuated BP response during and after stress was linked to blunted stress‐evoked increases in peripheral vascular resistance with prazosin in young normotensive Black adults. Moreover, urinary sodium excretion rate, in response to stress, was not altered by prazosin treatment, which suggests that increased stress‐mediated renal sodium reabsorption did not contribute to the heightened BP response during stress in this study population. The results of this study support the role of α_1_‐adrenergic activation in stimulating and maintaining elevated BP levels in response to mental stress via increased peripheral vasoconstriction in Black adults.

The prevalence of HTN is higher in Black individuals than any other racial group in the United States (Howard et al., 2013; Writing Group M, 2016). Accumulating evidence suggests that rather than inherent genetic differences (Zilbermint et al., 2019), social and environmental factors including neighborhood crime, sleep quality, socioeconomic status, and access to healthcare play a major role in the racial disparity in the prevalence and severity of HTN (Calhoun, 1992; Cene et al., 2009; Curtis et al., 2017; Mujahid et al., 2011). Indeed, the concept that Black Americans are at higher risk for hypertension due to selection for salt retention, termed the “slavery hypertension hypothesis” is problematic and largely unsupported (Lujan & DiCarlo, 2018). In addition to a greater level of stress reported in Black individuals compared to other groups (Calhoun, 1992), Black individuals have been shown to have exaggerated pressor responses to acute stressors (Light et al., 1993; Murphy et al., 1991). Our 45‐min mental stress protocol elicited an average increase of 6.0 mmHg for SBP and 3.7 mmHg for DBP mmHg with placebo, while the magnitude of this increase was blunted (3.6 mmHg for SBP and 2.5 mmHg for DBP) with 7 days of prazosin treatment. Importantly, this prazosin‐induced dampening of BP reactivity was extended during the recovery period. Augmented pressor response to stress has been linked to increased risk for the development of HTN in healthy normotensive adults, including Black adults (Markovitz et al., 1998; Steptoe & Marmot, 2005). Furthermore, the efficient recovery of BP after stress, in terms of the timing and the magnitude of the drop back to pre‐stress levels, has been associated with lower HTN risk (Schneider et al., 2003; Schuler & O'Brien, 1997). In the current study, SBP reached resting levels at the beginning of the recovery period, and in fact reached levels significantly lower than resting levels 30 min into recovery (113 versus 116 mmHg, *p* = .01) with prazosin treatment. Moreover, with placebo treatment, SBP was still significantly elevated at the beginning of the recovery compared to rest and remained higher than the resting value throughout the recovery period. Taken together, our results suggest that α_1_‐adrenergic activation plays an important role in stress‐associated BP regulation and inhibition of α_1_‐adrenergic receptors activity attenuates BP reactivity to stress, as well as induces efficient BP recovery after stress in normotensive Black adults.

The increase in BP during stress was accompanied by an increase in TPR with placebo, while there was no increase in TPR during stress with prazosin treatment. Instead, there was a greater increase in cardiac response (i.e., HR and CO) during stress with prazosin compared to placebo. The results with placebo are consistent with previous findings demonstrating an exaggerated increase in vascular reactivity to stressors (Adefurin et al., 2013; Sherwood & Hinderliter, 1993; Stein et al., 2000), which was linked to increased pressor responses in normotensive Black individuals (Okada et al., 2016; Stein et al., 2000). Our findings provide a mechanistic insight for the previous findings by demonstrating that the BP increase, in response to an acutely prolonged mental stress, is in part mediated by α_1_‐adrenergic action on peripheral vascular resistance in Black adults. It is well established that the activation of SNS releases catecholamines that cause vascular smooth muscle contraction by activating α_1_‐adrenergic receptors. Previous studies indicate a prominent role of α_1_‐adrenergic receptor‐mediated vasoconstriction in the stress response in high‐risk populations (Adefurin et al., 2017; Sherwood et al., 2017). For example, increased vascular α_1_‐adrenoreceptor sensitivity as demonstrated by an exaggerated vasoconstrictive response to an α_1_‐ adrenoreceptor agonist has been previously described in Black individuals (Adefurin et al., 2013; Stein et al., 2000). Our results with placebo showed that, in addition to an immediate and maintained elevation in TPR during stress, TPR was still above the baseline throughout the recovery period, while cardiac variables were back to the baseline upon the cessation of stressor. This may indicate a lasting effect of increased sympathetic activation on peripheral vasoconstriction during the 45‐min Recovery period. Several factors are suggested to contribute to exaggerated sympathetic vascular transduction in Black individuals, such as elevated increase in vasoconstriction in response to a given increase in sympathetic neural outflow (Vranish et al., 2018), including elevated oxidative stress, the subsequent reduction in nitric oxide bioavailability (Mata‐Greenwood & Chen, 2008) and blunted β_2_–adrenoreceptor‐mediated vasodilation (Mehta et al., 1995; Stein et al., 2000). Prazosin treatment, moreover, led to a blunted increase in TPR during stress. This may explain the observations of greater stress‐induced increases in HR, SV, and CO as a counterregulatory stress response, as well as the positive association between HR and BP during stress with prazosin treatment. In fact, this greater involvement of CO as a part of cardiovascular stress response with prazosin treatment in this study mirrors the hemodynamic action seen in White individuals, who are known to have greater cardiac control during stress, compared to Black individuals (Okada et al., 2016). Interestingly, we found a positive association between TPR and BP during the recovery period with both treatments, which may indicate that the BP recovery process is determined as a function of post‐stress vascular tone change. Particularly, α_1_‐adrenoreceptor mediated‐vasoconstriction may have a lasting damaging effect in the context of repeated episodes of stress even in young healthy Black adults which in the long run may contribute to tonic elevations in BP.

Another important source of hemodynamic regulation, during and after stress, is sodium handling via increased renal sympathetic activation (Johns et al., 2011; Schlaich et al., 2009). This may be especially relevant given that a large proportion of Black individuals are described as having a dysfunctional BP response to salt intake, that is, salt sensitivity (Svetkey et al., 1996) although an alternative view is recently raised that salt sensitivity may not be a direct function of sodium retention within cohorts of Black adults (Kurtz et al., 2016; Morris et al., 2016). Our previous findings indicate that salt sensitivity can be induced by mental stress in some Black adults with impaired sodium excretory capacity in response to stress‐induced increases in BP (Harshfield, et al., 2002). Therefore, we hypothesized that the activation of renal SNS is responsible for stress‐associated sodium retention in Black adults and the sodium excretion rate, in response to stress, would be greater with prazosin treatment compared to placebo. In contrast to our hypothesis, the stress‐associated sodium excretion rate was similar between prazosin and placebo treatments. There are potential methodologic issues that may have decreased the accuracy of the urinary sodium excretion measurements including spontaneously voided urine collection rather than using a Foley catheter. However, our previous studies have suggested that this urinary sodium collection protocol is valid and sensitive for detecting changes in response to stress (Barbeau et al., 2003; Harshfield, et al., 2002), and detecting group differences (Harshfield, et al., 2002; Harshfield et al., 2003, 2007), and treatment effects (Jeong et al., 2019) in stress‐associated urinary sodium excretion. Although both subtypes of α_1_‐ and α_2_‐adrenergic receptors are involved in sodium reabsorption at the level of the proximal tubules, α_2_‐adrenergic receptors also mediate diuresis at the collecting duct via antidiuretic hormone (Pettinger et al., 1987) and renal α_2_‐adrenergic receptor sensitivity increases in a compensatory manner following α_1_‐adrenergic receptor blockade (Jeffries et al., 1987). In addition, animal evidence suggests that pathological sodium retention is linked to salt‐induced HTN, which may be mediated by renal α_2_‐adrenergic receptors (Makaritsis et al., 1999). Therefore, although not directly tested, the net sodium excretion, in response to stress, may not have been altered due to a dominant and/or compensatory action of other adrenergic mechanisms, such as α_2_‐adrenergic receptor‐mediated sodium reabsorption. Alternatively, non‐adrenergic pathways, such as the renin‐angiotensin system, may have been involved in stress‐associated sodium dysregulation in Black adults (Jeong et al., 2019), likely in a dependent manner with adrenergic mechanisms masking the inhibitory effect of α_1_‐adrenergic receptor blockade on renal sodium retention. Therefore, the complex neurogenic and non‐neurogenic interactions should be considered when investigating mechanisms of renal sodium regulation in the setting of genetically or environmentally increased renal sympathetic activation.

The attenuation in BP and vasoconstriction with prazosin was only observed during and after stress, but not at rest. The lack of reduction in resting BP may be due to the normotensive status of the participants, the low dose (1 mg/day), and short duration (7 days) of drug treatment. This similar resting BP at both testing sessions, however, is the strength of the study in that it allowed us to evaluate the effects of α_1_‐blockade in stress reactivity, without the confounding effects of changes in resting BP. A limitation of the study is the lack of a direct measurement of sympathetic activation (i.e., microneurography or circulating catecholamine levels) or perceived stress levels. However, resting sympathetic nerve activation measured by microneurography (Fonkoue et al., 2018) and norepinephrine spillover (Stein et al., 2000) and sympathetic response to lower body negative pressure and cold pressure test in local and circulating norepinephrine spillover in Black individuals has previously been shown to be similar to other racial groups (Stein et al., 2000). Thus, we speculate that the basal sympathetic activation of normotensive Black adults is likely to be in a normal range, which may explain the minimal influence of prazosin on basal vasoconstriction. Next, there may be potential variation in sympathetic outflow in some female participants whose visits were in different phases of their ovarian cycle. However, although sympathetic activity fluctuates with hormonal changes during the ovarian cycle, transduction of sympathetic activity into vascular resistance is independent of the ovarian cycle (Minson et al., 2000). Furthermore, the intra‐individual variability of SBP, HR, and TPR between both sessions, in female participants, was not significant and similar to that of male participants. Given the known salt‐sensitivity in Black individuals, the sodium‐controlled diet employed prior to the experimental days may have affected the BP response to stress if participants’ typical salt intake range was different from the level used in this study. Considering the level used in this study (4,000 mg/day) falls in the high end of the average sodium intake in the general U.S. population (Jackson et al., 2016), introduction of increased salt intake might have occurred during the study period in some participants, which could facilitate increased pressure response to a stressor via impaired vasodilatory function and exaggerated sympathetic outflow (Babcock et al., 2020; Baric et al., 2019). Similar to our previous study (Jeong et al., 2019), a relatively modest magnitude of stress pressor reactivity was observed in this study (6.0 ± 8.9 mmHg with placebo), compared to other studies using different types of mental stressors (> 10 mmHg) (Light et al., 1993; McAdoo et al., 1990) or a video games stress test that lasted a few minutes (~10 mmHg) (Markovitz et al., 1998; Murphy et al., 1986) in Black individuals, although a direct comparison may not be applicable due to the different types and length of the mental stressor. Last, the type of mental stress we employed (a competitive video game against another individual for monetary reward) unlike memory‐driven or emotional arousal‐evoking tasks, is likely to elicit a similar magnitude of sympathetic activation during separate testing sessions within the same individual. Nevertheless, the collection of perceived stress levels at each stress testing session would be informative to potentially adjust for the possible discrepancy in the perceived stress levels between sessions.

## 5 CONCLUSION

Our results indicated that the increased pressor response to mental stress and delayed recovery process occur, in part, via a neurogenic vascular mechanism mediated by α_1_‐adrenergic receptors in Black adults. We demonstrated that α_1_‐adrenergic receptors blockade with prazosin led to reduced BP increases during prolonged mental stress and improved the post‐stress BP during the recovery phase, which was linked to reduced vasoconstriction, but without changes in renal sodium excretion in young normotensive Black adults. The results of this study may provide insight into the pathophysiology of stress‐associated HTN and may also inform the development of treatment targets to prevent the development and progression of HTN in Black adults.

## CONFLICT OF INTEREST

No conflicts of interest, financial or otherwise, are declared by the author(s).

## AUTHOR CONTRIBUTIONS

J.J., G.K., and G.A.H. conceived and designed research; M.L.B. and G.A.H. performed experiments; J.J., G.A.H., and J.P. analyzed the data; J.J, M.L.B., G.K., G.A.H., and J.P. interpreted the results of experiments; J.J. prepared figures; J.J., M.L.B., G.A.H., and J.P. drafted manuscript; J.J, M.L.B., G.K., G.A.H., and J.P. edited and revised manuscript; J.J, M.L.B., G.K., G.A.H., and J.P. approved final version of the manuscript.
